# Combination chemotherapy with mitomycin C and methotrexate is active against metastatic HER2-negative breast cancer even after treatment with anthracycline, taxane, capecitabine, and vinorelbine

**DOI:** 10.1186/s40064-015-1159-4

**Published:** 2015-07-26

**Authors:** Takayo Fukuda, Masahiko Tanabe, Kokoro Kobayashi, Ippei Fukada, Shunji Takahashi, Takuji Iwase, Yoshinori Ito

**Affiliations:** Breast Oncology Center, Cancer Institute Hospital, Japanese Foundation for Cancer Research, Koto-ku, Tokyo Japan; Department of Translational Oncology, St. Marianna University Graduate School of Medicine, Kawasaki, Kanagawa Japan; Department of Breast Oncology, Juntendo University School of Medicine, Bunkyo-ku, Tokyo Japan

**Keywords:** Metastatic breast cancer, Methotrexate, Mitomycin C, Capecitabine, Vinorelbine, Anthracycline, Taxane

## Abstract

**Background:**

Combination chemotherapy with mitomycin C and methotrexate (MM) was reported to be effective for 24% of patients with metastatic breast cancer (MBC) who had been treated with anthracycline and taxane. Antimetabolites such as capecitabine and antitubulins such as vinorelbine have been generally used for MBC treatment after anthracycline and taxane. A subsequent choice of chemotherapy should be offered to patients with MBC who have kept good performance status (PS) after being aggressively treated with anthracycline, taxane, capecitabine, and vinorelbine (ATCV), but is not well clear which treatment is superior to others after ATCV. In this study, we examined whether MM treatment is a good choice following ATCV.

**Methods:**

We retrospectively reviewed the medical records of 31 patients with HER2-negative metastatic breast cancer who were treated with MM following ATCV. One cycle of MM was defined as MMC 8 mg/m^2^ on day 1 and MTX 60 mg/m^2^ on day 1 and day 15, administered intravenously every 4 weeks.

**Results:**

Response rate and clinical benefit rate were 9.7 and 19.4%, respectively. Median times to progression and times to failure were 3.9 and 3.7 months, respectively. Adverse events of grade 3 and/or 4 were observed in 36% patients. Thrombocytopenia of grade 3 or 4 was 12.9 and 3.2%. Grades 3 and 4 of leucopenia and anemia were 12.9 and 9.7%, respectively.

**Conclusion:**

MM is effective and tolerable for MBC patients even after aggressive treatment with ATCV. MM is one treatment choice when patients have kept good PS and bone marrow function even after multiple regimens of chemotherapy.

## Background

Treatment for breast cancer generally has two aspects. One is surgery for local control; the other is systemic treatment to limit or eliminate potentially metastatic disease. Sequential administration of anthracycline and taxane is recommended as systemic chemotherapy in neo-adjuvant or adjuvant settings, depending on risk factors such as metastasis in axillary lymph nodes. Once metastatic breast cancer (MBC) has been diagnosed with radiological or imaging assessments, it is very difficult to achieve complete eradication of MBC while maintaining both the length and the quality of patients’ lives.

Cytotoxic drugs playing major roles in treating MBC include antitubulins such as vinorelbine and eribulin, antimetabolites such as fluorouracil derivatives, capecitabine or S-1, and gemcitabine.

With vinorelbine, the recurrence rate (RR) for advanced or recurrent breast cancer previously treated with anthracycline and taxane was 20–25% (Toi et al. 2005; Livingston et al. 1997; Zelek et al. 2001), and TTP was 91–115 days (Toi et al. 2005; Livingston et al. 1997).

With eribulin for locally recurrent or metastatic HER2 negative breast cancer, RR was 13–29%, CBR was 23–52%, and the median progression-free survival (PFS) was 3.7–6.8 months (McIntyre et al. 2014; Aogi et al. 2012; Cortes et al. 2011).

With Capecitabine for MBC patients who had failed with a regimen containing anthracycline and taxane, median RR was 23.6% (15–29%). Median TTP was 96.7 days (89–107 days) (Blum et al. 1999; Blum and Dieras 2001; Reichardt et al. 2003; Fumoleau et al. 2004; Wist et al. 2004).

In S-1 for 35 MBC patients pretreated with anthracycline, taxane, and capecitabine, RR was 3%, and the clinical benefit rate (CBR) was 20%. TTF was 2.8 months (Ito et al. 2009).

Although MBC can be resistant to treatments that include anthracycline, taxane, capecitabine, and vinorelbine, a substantial number of patients have kept good performance status (PS). Such patients are eager for the next effective treatment to keep their MBC under control and to maintain their quality of life.

We previously reported that the combination therapy of MMC and MTX (MM) was effective for MBC patients pretreated with anthracycline and taxane (Tanabe et al. 2009). Partial response (PR) was observed in 24% patients, and TTP was 4.8 months. We hypothesized that this combination treatment would have the potential to control MBC in appropriately selected patients. When PS is good even after anthracycline, taxane, capecitabine, and vinorelbine, patients may be able to tolerate subsequent chemotherapy.

Here, we report a retrospective analysis of the activity of MMC and MTX for HER2-negative patients with MBC who had been treated with ATCV.

## Methods

### Patients

We reviewed the medical records of patients whose MBC had been treated with MM from September 2005 to July 2007 at a Cancer Institute Hospital. The eligibility criteria were as follows: (1) clinically and histologically confirmed MBC; (2) prior treatment with anthracycline, taxane, capecitabine, and vinorelbine; (3) absolute neutrophil count >1,500 μL; (4) transaminase <2.5 × UNL (in case of hepatic metastasis, <5 × UNL); (5) serum creatinine <1.5 × UNL; (6) measurable lesion(s) according to the Response Evaluation Criteria in Solid Tumor (RECIST) guidelines Ver.1.1; (7) performance status of 0 or 1 based on the Eastern Cooperative Oncology Group scale; (8) written informed consent from each patient.

### Administration schedule of MM

MMC 8 mg/m^2^ was intravenously given on day 1 and MTX 60 mg/m^2^ on day 1 and day 15 every 4 weeks. After MMC reached a cumulative dose of 50 mg/m^2^, only MTX of 60 mg/m^2^ on day 1 and day 15 was repeated until progressive disease (PD) or adverse events were observed.

When hematological toxicity of grade 3 or 4 was observed, dosage was reduced by 20% in the next treatment. When hematological or other toxicity was grade 2 or 3, the treatment date was postponed for 1 week or more until patients recovered from the toxicity. When the performance status became worse than grade 3, the treatment was stopped and changed. Adverse events were graded by Common Terminology Criteria for Adverse Events (CTCAE) v3.0. Symptomatic adverse events were evaluated based on questionnaires filled out by patients when they visited our hospital and confirmed by the physician’s interview and medical examination.

### Evaluation of efficacy and safety

All patients were followed up with hematological findings, chest-abdominal CT, bone scintigraphy, and ultrasonography according to metastatic sites.

Responses were assessed according to the RECIST v1.1. Complete response (CR): Disappearance of all target lesions. Any pathological lymph node (whether target or non-target) must have reduction in the short axis to <10 mm. Partial response: At least a 30% decrease in the sum of diameters of target lesions, taking as a reference the baseline sum diameters. Progressive disease: At least a 20% increase in the sum of diameters of target lesions, taking as a reference the smallest sum while in the study (including the baseline sum if that is the smallest). In addition to the relative increase of 20%, the sum must also demonstrate an absolute increase of at least 5 mm. The appearance of one or more new lesions is also considered progression. Stable disease (SD): Neither sufficient shrinkage to qualify for PR nor sufficient increase to qualify for PD, taking as a reference the smallest sum diameters while in the study (Eisenhauer et al. 2009). Objective response rate (ORR) was defined as the sum of the CR and PR rates; CBR was defined as the sum of the CR, PR, and long SD rates. TTP was defined as the period from the beginning of the first cycle of MM treatment to PD, and TTF was defined as the period from the initial MM treatment to its discontinuation because of PD or unacceptable toxicity. All adverse events and laboratory parameters were graded according to CTCAE v 3.0.

### Statistical analysis

TTP and TTF were calculated by the Kaplan–Meier method, using SPSS ver. 17. (IBM, Japan).

## Results

### Patient characteristics

The patients’ characteristics are shown in Table 1. Thirty-one patients received MM between September 2005 and July 2007. Their median age was 53 years (range 30–75). All patients were Japanese; 30 were female and one was male. Twenty-nine patients (93.5%) had a performance status score of 0. Fifteen patients (48.4%) were positive for both of estrogen receptor (ER) and progesterone receptor (PgR), six (19.4%) were ER positive and PgR negative, and 10 (32.3%) were negative for both. Nineteen patients (61.3%) had more than three metastatic sites. All patients had been previously treated with ATCV. They received the MM regimen as the 5–8th line (median 5) treatment (Table 1).

**Table 1:** Patient characteristics (n = 31)

Characteristics	Patients, n	%
Median age (range)	53 (30–75)	
Male	1	3.2
Female	30	96.8
Performance status		
0	29	93.5
1	2	6.5
ER/PgR status		
Positive/positive	15	48.4
Positive/negative	6	19.4
Negative/negative	10	32.3
No. of metastasis		
Median (range)	3 (1–5)	
1	5	16.1
2	7	22.6
3	15	48.4
4	3	9.7
5	1	3.2
Site of metastasis		
Lymph node	16	51.6
Chest wall/skin	3	9.7
Lung	16	51.6
Pleura	3	9.7
Bone	18	58.1
Liver	18	58.1
Contra late ral breast	1	3.2
Muscle	1	3.2
Peritoneum	2	6.5
No. of prior chemotherapy		
Median (range)	5 (5–8)	
5	19	61.3
6	9	29.0
7	1	3.2
8	2	6.5
Agent used in prior chemotherapy		
CMF	4	12.9
Anthracycline	31	100
Taxane	31	100
Paclitaxel	11	35.5
Docetaxel	26	83.9
Capecitabine	31	100
Vinorelbine	31	100
S-1	1	3.2
UFT	3	9.7
5-DFUR	1	3.2
Other	1	3.2

*ER* estrogen receptor, *PR* Progesterone receptor, *CMF* cyclophosphamide, methotrexate, and fluorouracil.

### Efficacy

**Fig. 1:**
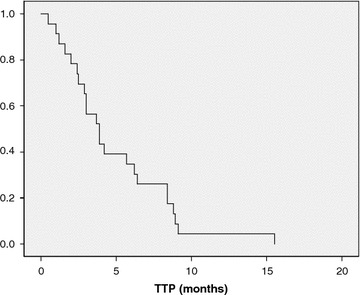
Kaplan–Meier time to progression (TTP) curve. Median TTP was 3.9 months (95% CI 2.5–5.3) (n = 23).

**Fig. 2:**
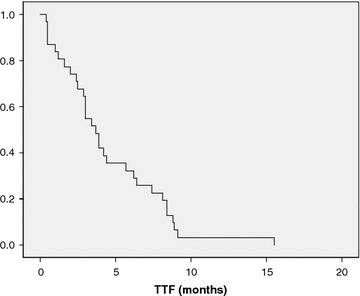
Kaplan–Meier time to failure (TTF) curve. Median TTF was 3.7 months (95% CI 2.9–4.5) (n = 31).

Twenty-three patients (74.2%) were able to continue MM until PD was observed. Eight patients (25.8%) terminated MM because of adverse events. They received the MM therapy for 1–13 cycles with a median of 4.6 cycles; CR was not observed. Three patients (9.7%) achieved PR. Nine patients (29%) had SD. Three patients (9.7%) maintained stability for more than 24 weeks. RR and CBR were 9.7 and 19.4%, respectively (Table 2). The median TTP was 3.9 months (Fig. 1). TTF was 3.7 months (Fig. 2).

**Table 2:** Response rate (n = 31)

Response	n	%
Complete response	0	0
Partial response	3	9.7
Stable disease	6	19.4
Long stable disease	3	9.7
Progressive disease	19	61.3
Objective response rate	3	9.7
Clinical benefit rate	6	19.4

In luminal-type breast cancer, one patient (4.8%) achieved PR, and six patients (28.6%) had SD. In triple negative breast cancer, two patients (20%) achieved PR, and three patients (30%) had SD.

### Safety

Grade 3 or 4 adverse events were seen in 11 patients (36%). Grade 3 thrombocytopenia was observed in four (12.9%) and Grade 4 in one (3.2%). Grade 3–4 leucopenia was observed in four (12.9%) and grade 3–4 anemia in three (9.7%). No patients had renal toxicity resulting from MM. All toxicity was manageable. Dose reduction was necessary in seven (22.6%) patients mainly due to hematological toxicity (Table 3). Renal toxicity, hemolytic uremic syndrome (HUS), was not experienced.

**Table 3:** Toxicity, patient’s number (n = 31)

Toxicity	G1	%	G2	%	G3	%	G4	%
Thrombocytopenia	5	16.1	1	3.2	4	12.9	1	3.2
Leucopenia	3	9.7	7	22.6	2	6.5	2	6.5
Anemia	9	29.0	5	16.1	1	3.2	2	6.5
AST/ALT elevation	4	12.9	1	3.2	2	6.5	0	0
Anorexia	0	0.0	3	9.7	1	3.2	0	0
Neuropathy	2	6.5	4	12.9	1	3.2	0	0
Nausea	2	6.5	3	9.7	0	0	0	0
Vomiting	3	9.7	1	3.2	0	0	0	0
Constipation	4	12.9	0	0	0	0	0	0
Diarrhea	5	16.1	0	0	0	0	0	0

*G1-4* CTCAE grade 1–4.

## Discussion

MM treatment yielded relatively good results as a late line of treatment for metastatic breast cancer even after heavy treatment with ATCV.

MMC, which acts as an alkylating agent, has been used in combination chemotherapy for various tumors since 1974. MMC binds to DNA to form an interstrand DNA–DNA cross-link, which is believed to cause its cytotoxic effect. MTX inhibits dihydrofolate reductase, which halts the cell cycle by decreasing the synthesis of thymidylate and purine nucleotide.

MTX is a folate analog designed to inhibit dihydrofolate reductase. Reduced folate is the proximal single carbon donor in several reactions involved in the de novo synthetic pathways for purine and pyrimidine precursors of DNA and RNA that are required for cell proliferation.

While new drugs are currently being developed, several conventional drugs such as MMC and MTX have become less often administered. But these useful drugs, well documented for efficacy and safety, have the potential to control MBC even after aggressive treatment with multiple regimens because MMC and MTX, respectively, have pharmacological mechanisms that are different from previously used anti-cancer drugs. When considering the biological function of anti-cancer action and cross-resistance, it has been speculated that drugs with the same pharmacological mechanisms as previously used drugs would not have enough power to control metastatic breast cancer after ATCV. Capecitabine and S-1 are known as prodrugs of Fluorouracil (FU) and were selectively activated by tumor cells enough to exert cytotoxic activity. Although they may seem to be newly administered drugs, fluorouracil derivatives such as FU have already been used with anthracycline regimens such as CAF (Cyclophosphamide, Adriamycin, and FU) or CEF (Cyclophosphamide, Epirubicin, and FU) in most MBC patients. Therefore, it is supposed that in some cases, resistance to FU might have been established before administration of capecitabine and S-1.

Vinorelbine binds to tubulin, inhibiting tubulin polymerization into microtubules. Spindle formation leads to apoptosis of cancer cells. Vinorelbine’s mitotic microtubule-inhibiting activity correlates with its antitumor efficacy (Toi et al. 2005). Eribulin, which is a non-taxane inhibitor of microtubule used as monotherapy (Cortes et al. 2011), binds to the vinca domain of tubulin and inhibits polymerization of tubulin and assembly of microtubules. In vinorelbine and eribulin treatment, inhibition of mitotic spindle assembly leads to cell cycle arrest at the G2/M phase. For eribulin, although the target points are different respectively, the target molecule itself is the same as in taxane and vinorelbine (Saji 2013; Andreopoulou and Sparano 2013).

MM therapy has a mechanism potentially different from other drugs previously used. In addition, combination chemotherapy has the potential to show clinical therapeutic efficacy compared to single-drug therapy (Andreopoulou and Sparano 2013).

As mentioned in the introduction, MM was effective for 24% of the MBC patients treated with anthracycline and taxane. The median TTP was 4.8 months when MM was used as third-line therapy (Tanabe et al. 2009).

There are studies reporting the clinical efficacy of treatments including MMC and MTX. In a combination of MMC, MTX, and VP-16 (VMM), the response rate was 31%, the clinical benefit rate 47%, the median disease-stabilization duration was 9.1 months, and the median PFS was 4.2 months (Aldabbagh 2009). Combination treatment with mitoxantrone, MMC, and MTX (MMM) was reported to be as effective as CMF. MMM achieved an objective response (51%) comparable to that with CMF (60%). Overall median survival was 16 months for MMM and 12 months for CMF (Jodrell et al. 1991). However, when cardiac toxicity is considered, administration of mitoxantrone cannot be recommended after treatment with anthracycline.

The clinical results in these studies support that MM shows strong potential as a treatment for MBC. Moreover, MM might be more effective than monotherapies such as capecitabine, vinorelbine, and eribulin (Andreopoulou and Sparano 2013).

Treatments for primary breast cancer as well as MBC are selected depending on the expression patterns of ER, PgR, and HER2. In luminal-type breast cancer, we have several choices of endocrine therapy such as selective estrogen receptor modulators, selective estrogen receptor down-regulators and aromatase inhibitors. In HER2 positive type breast cancer, we usually use anti-HER2 target therapy such as trastuzumab, lapatinib, pertuzumab with cytotoxic agents, or hormonal treatment. However, there is no promising treatment that is effective for triple negative (TN) breast cancer, defined as ER negative, PgR negative, and HER2 negative. Therefore, it is important to develop a variety of treatments to increase the choices of chemotherapy with different molecular mechanisms.

In our study, 10 patients were TN. Two out of the 10 had PR, 1 showed long SD, 2 had SD, and the median TTF was 4.4 months. Although it was a small number, CBR was 30%. On efficacy of eribulin as the third-line therapy in 22 TN patients, PFS was 1.8 months and RR was 13.8% (Aogi et al. 2012). Eribulin as 1st line therapy, 12 of TN patients were treated, RR was 16.7% CBR was 25%, respectively (McIntyre et al. 2014). Although our report was retrospective one, the results deserve attention in that all patients were pretreated with ATCV and MM was administered later than the fifth line. We found no report dealing with chemotherapy for patients with metastatic breast cancer, all of whom had been pretreated with four regimens of ATCV. Based on our results, MM is a potential choice of treatment as fifth-line therapy for MBC pretreated with ATCV.

Jia et al. developed new drug delivery systems of MTX and MMC loaded PEGylated chitosan nanoparticles (CS-NPs), which coordinate the early phase targeting effect with the late-phase anticancer effect (Jia et al. 2014). The (MTX + MMC)-PEG-CS-NPs exhibited concentration- and time-dependent cytotoxicity. Jia et al. showed that codelivery of MTX and MMC suppresses tumor cell growth to a greater extent than the delivery of either drug alone, indicating a synergistic effect. Synergizing the therapeutic index might be effective for patients to maximize the therapeutic effects of MMC while minimizing its toxicity. These encouraging data support our results.

## Conclusion

MM treatment may be both effective and tolerable for heavily treated patients with good performance status.

## Abbreviations

MMC: mitomycin C
MTX: methotrexate
MM: therapy combining MMC and MTX
MBC: metastatic breast cancer
CMF: cyclophosphamide/methotrexate/fluorouracil
CR: complete response
PR: partial response
SD: stable disease
PD: progressive disease
RR: response rate
CBR: clinical benefit rate
RECIST: response evaluation criteria in solid tumors
CTCAE: common terminology criteria for adverse events
PS: performance status
HER2: human epidermal growth factor receptor type 2
ER: estrogen receptor
PgR: progesterone receptor
TN: triple negative
TTP: time to progression
TTF: time to failure
